# New vistas unfold: Chicken MHC molecules reveal unexpected ways to present peptides to the immune system

**DOI:** 10.3389/fimmu.2022.886672

**Published:** 2022-07-29

**Authors:** Samer Halabi, Jim Kaufman

**Affiliations:** ^1^ Institute for Immunology and Infection Research, University of Edinburgh, Edinburgh, United Kingdom; ^2^ Department of Pathology, University of Cambridge, Cambridge, United Kingdom; ^3^ Department of Veterinary Medicine, University of Cambridge, Cambridge, United Kingdom

**Keywords:** avian, BF, BL, peptide-binding specificity, peptide-translocation specificity, evolution

## Abstract

The functions of a wide variety of molecules with structures similar to the classical class I and class II molecules encoded by the major histocompatibility complex (MHC) have been studied by biochemical and structural studies over decades, with many aspects for humans and mice now enshrined in textbooks as dogma. However, there is much variation of the MHC and MHC molecules among the other jawed vertebrates, understood in the most detail for the domestic chicken. Among the many unexpected features in chickens is the co-evolution between polymorphic TAP and tapasin genes with a dominantly-expressed class I gene based on a different genomic arrangement compared to typical mammals. Another important discovery was the hierarchy of class I alleles for a suite of properties including size of peptide repertoire, stability and cell surface expression level, which is also found in humans although not as extreme, and which led to the concept of generalists and specialists in response to infectious pathogens. Structural studies of chicken class I molecules have provided molecular explanations for the differences in peptide binding compared to typical mammals. These unexpected phenomena include the stringent binding with three anchor residues and acidic residues at the peptide C-terminus for fastidious alleles, and the remodelling binding sites, relaxed binding of anchor residues in broad hydrophobic pockets and extension at the peptide C-terminus for promiscuous alleles. The first few studies for chicken class II molecules have already uncovered unanticipated structural features, including an allele that binds peptides by a decamer core. It seems likely that the understanding of how MHC molecules bind and present peptides to lymphocytes will broaden considerably with further unexpected discoveries through biochemical and structural studies for chickens and other non-mammalian vertebrates.

## Introduction

The major histocompatibility complex (MHC) is a genomic region originally discovered as the primary genetic locus responsible for graft rejection, but now is known to encode the highly polymorphic classical class I and class II molecules that present antigenic peptides to play crucial roles in innate and adaptive immune responses (1). Over three decades of research into human and mouse MHC molecules have provided very clear models of how MHC molecules acquire, bind and present peptides to thymus-derived (T) lymphocytes and natural killer (NK) cells (2, 3). Much less is known about the MHC systems in other species, ranging from mammals outside of primates and rodents to cartilaginous fish (4). A variety of studies have examined the structures of class I molecules in rabbit (5), horse (6), cow (7), swine (8–11), bats (12, 13), brushtail opossum (a marsupial mammal) (14), duck (a bird) (15), the frog *Xenopus* (16), grass carp (a bony ray-finned fish, or teleost) (17) and nurse shark (a cartilaginous fish) (18), which have shown some interesting differences compared to humans and mice. In parallel, studies into genomic organisation, and antigen processing and peptide loading have revealed great variation as well (4, 19).

Most of what is known about the MHC and MHC systems outside of placental mammals comes from research into the domestic chicken, *Gallus gallus domesticus* (4, 20, 21). This effort has benefited from nearly 150 years of research in the service of animal husbandry and later the global poultry industry, as well as lines of investigation like vaccination, virology, embryology and development. In comparison to typical mammals, the chicken MHC is small, simple and arranged differently, so much so that an overall different strategy of using MHC molecules was perceived, one of strong genetic associations commonly found for infectious disease. On the other hand, the simplicity of the chicken MHC has also allowed a clear appreciation of at least one fundamental property of classical class I molecules that is in common with humans, that of a hierarchy of peptide repertoires, which led to the hypothesis of generalists and specialists. Even less well understood is the focus of chicken class II molecules on a few genes from a pathogen with over 100 genes, Marek’s disease virus (MDV).

This review focuses on structural studies of classical chicken MHC molecules, and on the crucial functional and biochemical results that underpin them. However, it should be pointed out that there have also been studies of so-called non-classical MHC molecules in chickens, with structures of two CD1 molecules and a YF molecule, all of which appear to bind hydrophobic non-peptide antigens (22–24), presumably lipids.

## Big differences in the MHC of chickens and typical mammals

The first unexpected observation in chickens was the strong genetic associations of the B blood group with resistance and susceptibility to the lethal tumours caused by the oncogenic herpesvirus, MDV (25). These associations were eventually shown to be due to the so-called BF-BL region (26), which was found to determine the polymorphic class I (BF) and class II (BL) molecules (27). Cloning and sequencing eventually identified the BF-BL region as the chicken MHC (28, 29), defined as the primary locus for rapid allograft rejection, graft-versus-host reaction, mixed lymphocyte reaction and presentation of antigens to T lymphocytes.

In addition, some genomic DNA clones with class I and class II B genes were eventually shown to come from the so-called Rfp-Y region, which is genetically unlinked to the B locus but on the same chromosome (30). A region of repeats was found to separate the B and Y loci (31, 32). It has been forcefully argued that both regions should be called the MHC, MHC-B and MHC-Y (33, 34). However, the MHC genes from the Y locus are non-classical, with the class I YF molecule being highly polymorphic but not able to bind peptides and the class II YLB genes being non-polymorphic (35, 36). Thus, the Y region might be considered part of the extended MHC, in the same way as the HFE gene in humans and the Q, TL and M regions of mice. Reports ascribe a variety of immunological phenomena to the Y region (37–39), but the graft rejection times are moderate (40, 41), much like collections of minor transplantation antigens in mice.

**Figure 1:**
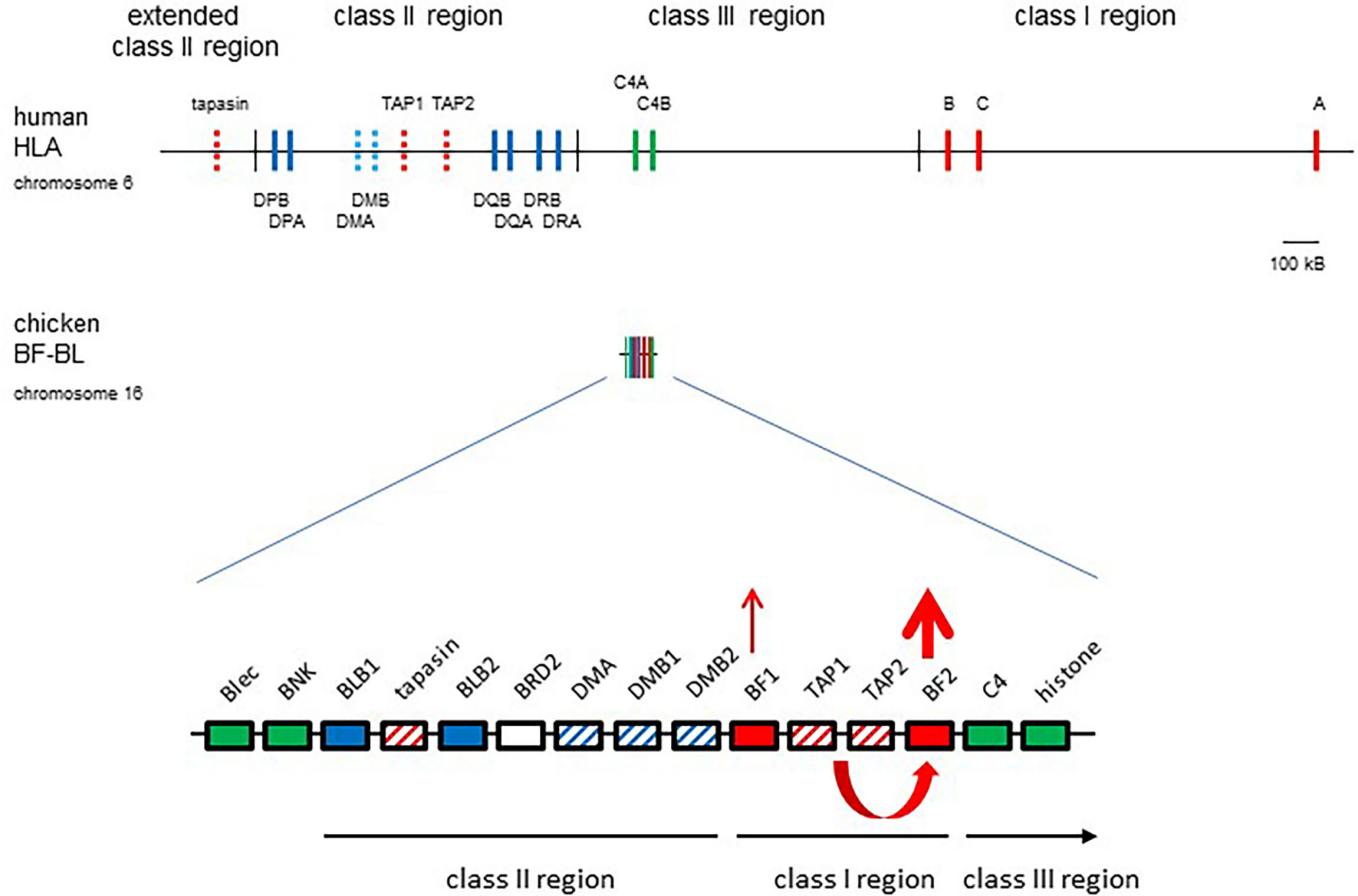
The chicken MHC (BF-BL region) is simpler and smaller than the human MHC (HLA), with co-evolution of polymorphic TAP1, TAP2 and tapasin genes with the BF2 gene leading to a single dominantly-expressed class I gene. Coloured boxes are genes with names above; thin vertical lines indicate region boundaries with names above or below; representation is roughly to scale, with a bar indicating approximately 100 kB. Gene expression indicated by thickness of arrows pointing up, co-evolution between TAP and BF2 genes shown by a curved arrow. Genes from the class I system, red; the class II system, blue; the class III or NK genes, green; solid colours indicate classical genes while striped colours indicate genes involved in peptide loading. Figure from reference (22).

Many features of the chicken MHC differ markedly from typical mammals (**Figure 1**), including the strong genetic associations with infectious disease mentioned above, the class III region on the outside of the class I and class II regions, the polymorphic TAP genes in between the two class I genes, a polymorphic tapasin (also called TAP binding protein, or TAPBP) gene nearby, the lack of an obvious tapasin binding site in TAP1, a polymorphic NK receptor/monomorphic ligand gene pair and a very low level of recombination across the region (20, 29). The discovery that only one of the two classical class I genes is well-expressed led to a unifying hypothesis (20, 42–44): that co-evolution between polymorphic peptide-loading genes and the closely-linked BF2 gene led to a single dominantly-expressed class I gene whose peptide motif determined immune response. In contrast, the enormous human MHC was considered to have nearly monomorphic antigen processing and peptide loading genes that acted as average best-fits for a distantly-located multigene family of classical class I genes, which together led to the lower genetic associations of the MHC with infectious diseases. The first peptide motifs in chickens, indeed outside of mammals, were the basis for these concepts and will be described next.

## Peptide-binding motifs, peptide-translocation specificities and structures for fastidious chicken class I alleles

Key to the first understanding of how the chicken MHC works were the knowledge of genetic associations for infectious diseases, the existence of relatively-inbred experimental chicken lines based on MHC serological typing, the relatively large size of chickens for isolation of cells and molecules, and the existence of monoclonal antibodies to chicken MHC molecules. Some or all of these factors have been missing from studies for most vertebrates outside of humans and mice; thus far, none of the structures mentioned for species outside of mammals and chickens are with natural ligands identified from MHC molecules on the surface of cells.

The monoclonal antibodies enabled isolation of class I molecules from blood (particularly erythrocytes), spleen and eventually virally-transformed chicken cell lines. Despite the clear evidence of two classical class I genes, only one unambiguous peptide motif was found for each of the B4, B12, B15 and B19 haplotypes (42). This finding prompted a closer look, which showed that the BF2 genes are far better expressed than the BF1 molecules at the level of RNA, protein and peptide motif (43, 45). Most of the chicken self-peptides (and motifs) are octamers, but are reminiscent of peptides from certain mammalian class I molecules (42, 43). The peptide motif from B12 was somewhat like mouse K^b^ and D^b^ molecules (simple hydrophobic anchors at peptide position 5 and 8), from B15 was somewhat like human HLA-B27 (basic residues at position 1, arginine at position 2 and tyrosine at position 8 or 9), and from B19 was also somewhat like human HLA-B27 (some basic residues at position 1, arginine at position 2 and a variety of hydrophobic amino acids at position 8). In contrast, B4 was entirely different from any class I molecule previously described, with acidic residues at peptide positions 2 and 5, and almost completely glutamic acid at position 8. Wire models of class I molecules were entirely consistent, with BF2 residues at potential contact positions with appropriate chemical properties to bind peptides with these motifs (43).

**Figure 2:**
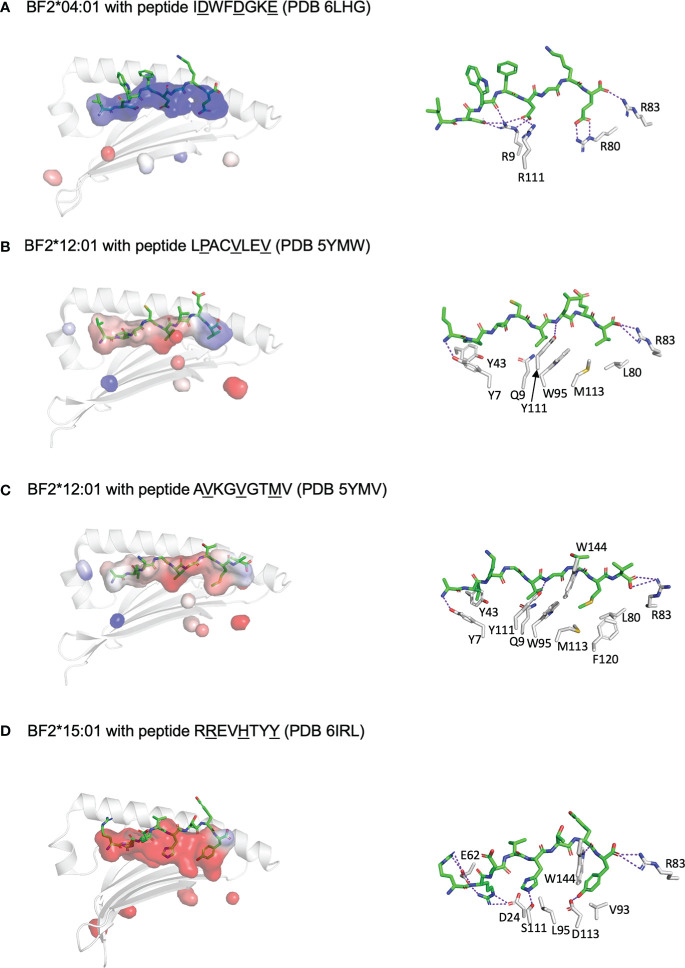
Three anchor positions are found for peptides bound to fastidious chicken class I molecules, which may have both fastidious and promiscuous pockets, and also illustrate C-terminal peptide extensions similar to peptides bound to class II molecules. Shown are peptides in sticks depiction and peptide-binding groove as surfaces with α1 helix and β-sheet in cartoon depiction (left panels) and with some key interactions between peptide and class I molecule (right panels). PyMol was used to determine the anchor residues (underlined in figure), the electrostatics for the class I surface (with electronegativity as red and electropositivity as blue), the presence of H-bonds and salt bridges between key class I residues and peptide side chains (as dotted purple lines), and some of the key class I residues within 4 Å of anchor residues. In addition, R83 is shown for each structure to show how it can allow C-terminal peptide extension. **(A)** BF2*04:01 with peptide IDWFDGKE (PDB 6LHG) has a largely basic groove, with pockets for anchor residues formed by Y7, R9, T24, Y43, Q62, I65, N69 and F97 for D2; R9, N69, I72, I73 and R111 for D5; and N76, I79, R80, R83, W95, T140 and W144 for E8. **(B)** BF2*12:01 with peptide LPACVLEV (PDB 5YMW) has a largely hydrophobic groove, with pockets for anchor residues formed by Y7, Y43, Q62, I65, Y97 and Y156 for P2; Q9, N69, I72, W95 and Y111 for V5; and N76, R83, M113 and K143 for V8; peptide position 2 is promiscuous despite being an anchor residue. **(C)** BF2*12:01 with peptide AVKGVGTMV (PDB 5YMV) as in panel B (but with the pocket around M8 being bounded by N76, I79, L80, W95, M113, F120, T140 and W144); R83 allows extension of peptide residue V9 out of the groove. **(D)** BF2*15:01 with peptide RREVHTYY (PDB 6IRL) has a largely acidic groove, with pockets for anchor residues formed by Y7, D24, T34, H35, E62, T65, S66 and Y97 for R2; I72, L95, Y97, S111 and Y149 for H5; and D73, T79, R83, V93, D113, T140, K143 and W144 for Y8; peptide position 1 points up out of the groove but is nearly always a basic residue interacting with E62, while position 5 is promiscuous despite being an anchor residue. Figure based on references (48–51).

More recently, structures of these molecules based on X-ray crystallography have become available (four for BF2*04:01, two for BF2*12:01, 4 for BF2*15:01) (46–49). These structures have confirmed all the inferences based on the motifs and the wire models, but with additional insights (**Figure 2**). For example, peptide position 2 for B12 is clearly an anchor residue embedded in the peptide-binding groove, but would not have been identified as an anchor residue based on the peptide motif, which fits with the notion of a “promiscuous pocket”. With this understanding, all chicken class I molecules for which there are structures suggest the presence of three anchor residues as defined by the side chains pointing down into the peptide-binding groove. As another example of a new insight, structures of BF2*04:01 and BF2*15:01 with CD8αα molecules showed two modes of interaction (49), one as found in mammals, but the other with a slightly different set of interactions, for reasons that are not yet clear.

Based on the two allelic lineages of TAP2 that co-evolve with class I alleles in rats (50, 51), chicken TAP and tapasin genes were examined and found to have enormous allelic polymorphism and moderate sequence diversity (44, 52). Peptide-translocation studies showed that the specificities were consonant with the BF2 peptide-binding specificities (44), and for two haplotypes, it was found that a narrower range of peptides was pumped compared to the range of peptides that could be refolded with BF2 molecules produced by bacterial expression (46, 53). The TAPs from B4 cells allowed the unprecedented translocation of peptides ending in an acid residue (44); chickens lack inducible proteasome components that in mammals favour peptides ending with hydrophobic or basic residues (54). Inspection of the protein sequences suggested obvious contact sites within the TAP1 and TAP2 alleles that might interact appropriately with peptides, suggesting the location of the peptide-binding sites (44, 55). However, comparison of the peptide-translocation specificities for cells with B12, B15 and B19 (which is a recombinant of B12 and B15) shows that variation within the TAP2 nucleotide-binding domain can affect peptide-translocation specificities. Sadly, the mutagenesis of chicken sequences for confirming these assignments, as well as understanding the single interaction of chicken TAP and tapasin, have never been finished, so there remains much to do in this area.

In a sense, the class I molecules in humans and mice take whatever peptides that they can bind from the wide variety provided by their TAPs, while these chicken class I molecules take what peptides they can get (56). In humans and mice, there are only a few TAP and tapasin alleles, only a few residues that vary, and no obvious functional differences. By contrast, the chicken TAP and tapasin alleles have moderate sequence diversity and are typically unique to each chicken MHC haplotype (44, 52, 57). The correlation of stringent peptide-translocation and peptide-binding specificities for TAP and class I alleles for these MHC haplotypes contrasts with monomorphic and wide peptide-translocation specificity of the nearly monomorphic TAPs and the multigene families of classical class I molecules in humans and mice. Indeed, the peptide-binding specificities of the chicken class I molecules from these haplotypes are generally more stringent than human class I alleles, leading to the label “fastidious”. A similar situation is expected for the alleles of the highly polymorphic tapasin, although there is functional evidence for only two haplotypes (57) and the obvious mutagenesis experiments are not finished.

## Peptide-binding motifs, peptide-translocation specificities and structures for promiscuous chicken class I alleles

A simple model for function of the chicken MHC arose from these first experiments: polymorphic TAP (and tapasin) genes co-evolve with only one of the two class I genes, so that a single dominantly-expressed class I molecule determines the cytotoxic T cell response, determining resistance or susceptibility which reads out as strong genetic associations (20, 42). In support of this view, the number of pathogen peptides predicted by peptide motifs for the B4, B12 and B15 BF2 alleles correlate well with the susceptibility to tumours induced by Rous sarcoma virus (RSV), and vaccination by the major binding peptide led to strong reduction of such tumours in B12 chickens (42, 43, 58). Similarly, a molecular-defined vaccine for infectious bursal disease virus (IBDV) showed the same correlation, with the numbers of peptides predicted and bound *in vitro* assembly assays correlating with response (59). However, the haplotypes with fastidious alleles like those in B4, B12, B15 and B19 generally are considered to confer susceptibility rather than resistance to the lethal tumours induced by MDV (25), historically one of the most important infectious pathogens.

Decades of experiments had identified B21 and B2 as the haplotypes that confer resistance to Marek’s disease, and some authorities summarised the historic literature to show a hierarchy of haplotypes, ranging from the most resistant B21 and B2 to the most susceptible B19 (25). Many experiments to determine the peptide motif for the class I molecules from B21 chickens found far fewer peptides and no simple peptide motif compared to the fastidious class I molecules (60). Moreover, flow cytometry of erythrocytes with the monoclonal antibodies to chicken class I and β_2_-microglobulin showed a hierarchy from low-expressing B21 to high-expressing B4, B15, B12 and B19, with the same rank order described for MDV resistance (42, 61). The existence of a cell-surface expression polymorphism among BF2 alleles, unprecedented for MHC molecules at the time, led to many experiments to test the hypothesis that NK cells were involved. This hypothesis seemed appropriate given the presence of an NK receptor gene (BNK) in the chicken MHC (29). However, eventually this family of NKR-P1 receptors in humans and mice was found to recognise lectin-like ligands encoded by neighbouring genes, which suggested the neighbouring lectin-like gene Blec (or other Blec-like genes in the BG region) as potential ligands for BNK (62–64).

The realisation that the expression level polymorphism was only part of a suite of properties for BF2 alleles came initially from biochemical experiments on the road to express the proteins for X-ray crystallography (60). The self-peptides identified for class I molecules from B21 cells had no positions with obviously similar residues, which led to the idea that several class I molecules with different motifs were responsible. However, expression of the BF2*21:01 heavy chain in bacteria followed by refolding with β_2_-microglobulin and different peptides *in vitro* showed conclusively that the same class I molecule could bind peptides with significantly different sequences.

**Figure 3:**
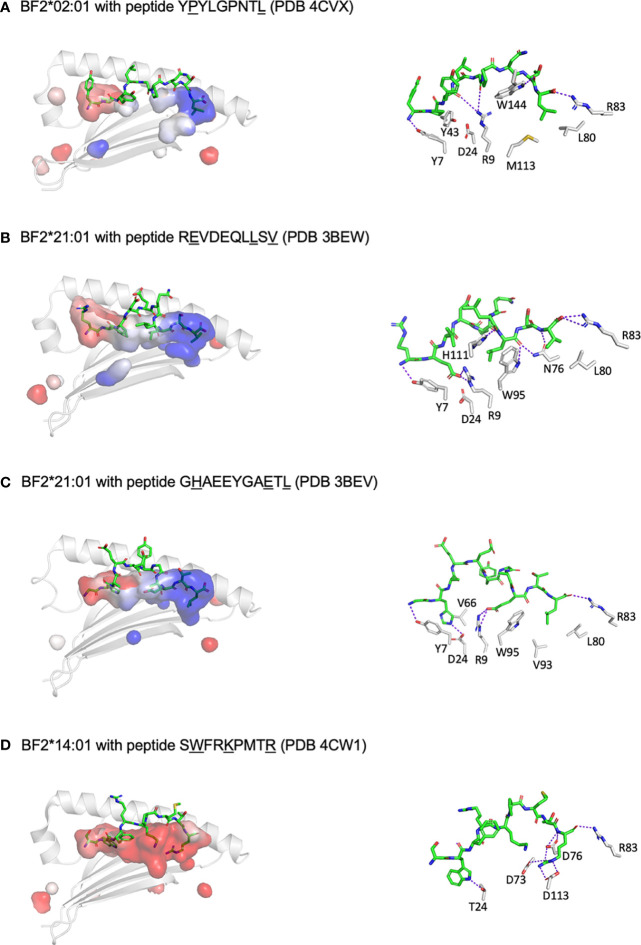
At least three different mechanisms allow a wide repertoire of peptides bound to promiscuous chicken class I molecules, including hydrophobic pockets (B2 and B14), remodelling binding site (B21) and C-terminal peptide extension (shown for B12 in **Figure 2**). Shown are peptides in sticks depiction and peptide-binding groove as surfaces with α1 helix and β-sheet in cartoon depiction (left panels) and with some key interactions between peptide and class I molecule (right panels). PyMol was used to determine the anchor residues (underlined in figure), electrostatics for the class I surface (with electronegativity as red and electropositivity as blue), the presence of H-bonds and salt bridges between key class I residues and peptide side chains (as dotted purple lines), and some of the key class I residues within 4 Å of anchor residues. In addition, R83 is shown for each structure to showcase C-terminal peptide extensions for some molecules. **(A)** BF2*02:01 with peptide YPYLGPNTL (PDB 4CVX) has two parts to the groove, the left being acidic and the right basic, with pockets for anchor residues formed by Y7, Y43, Q62, I65, Y97 and Y156 for P2; and N76, I79, L80, R83, M113, T140, K143 and W144 for L9; class I residues D24 and R9 do not interact with peptide side chains due to the relatively narrow groove, and peptide position 5 cannot be an anchor residue since G5 has no side chain (while another structure 4D0D has A5 in this position which does point down into the groove). **(B)** BF2*21:01 with peptide REVDEQLLSV (PDB 3BEW) has a groove with the left being acidic and the right basic, with pockets for anchor residues formed by Y7, R9, D24, E62, V66 and Y156 for E2; S69, I72, N73, N76, W95 and H111 for L8; and N76, L80, R83, T140 and K143 for V10; small class I residues G68, S69, S97 and G152 form a bowl to allow D24 and R9 to interact with anchor residue E2 by charge transfer. **(C)** BF2*21:01 with peptide GHAEEYGAETL (PDB 3BEV) as in panel B, but pockets for anchor residues including in addition M34, R61 and I65 but not R9 for H2; R9 and H111 for E9; and I79, V93, W95, F120 and V121 but no K143 for L11. **(D)** BF2*14:01 with peptide SWFRKPMTR (PDB 4CW1) has an acidic groove, with pockets for anchor residues formed by Y7, T24, V34, V43, Q62, I65, V66 and Y97 for W2; N69, I72, L95 and W144 for K5; and D73, D76, R83, V93, D113, T140 and W144 for R9; peptide position 5 is promiscuous despite being an anchor residue. Figure based on references (62, 63).

The mystery of such promiscuous binding was resolved by the X-ray crystal structures (six for BF2*21:01, two for BF2*02:01 and one for BF2*14:01) (60, 61), revealing unprecedented properties for class I molecules. BF2*21:01 binds peptides with three anchor residues (P_2_, P_c-2_ and P_c_) but remodels the peptide-binding site to accommodate a wide variety of co-varying amino acids at P_2_ and P_c-2_, based on four amino acid positions in the BF2 sequence (**Figure 3**). One small amino acid from each of the α-helices (serines at positions 70 and 99, using HLA-A2 numbering) together lead to a wide bowl in which an arginine at position 9 and an aspartic acid at position 24 pointing up from the underlying β-sheet have considerable conformational flexibility. Charge transfer between Arg9 and Asp24 allow interactions between acidic residues in the peptide and class I molecule, with the peptide positions P_2_ and P_c-2_ co-varying to accommodate the changes. A comparison between the original two self-peptides illustrates this idea. For the 11mer peptide GHAEEYGAETL, the basic His at P_2_ interacts with the acidic Asp24, while the acidic Glu at P_c-2_ interacts with Arg9. In contrast, for REVDEQLLSV, Glu at P_2_ directly interacts with the Asp24 that is interacting with Arg9; the movement of Arg9 creates a hydrophobic pocket for interaction with Leu at P_c-2_. A detailed analysis of the peptides bound *in vitro* by refolding peptide libraries and those found on the surface of cells by immunopeptidomics reveals that at least 50% of possible combinations of P_2_ and P_c-2_ can be found in the data. However, there are only a few combinations that are found at high frequencies.

There is a second mechanism by which chicken class I molecules can have a wide peptide repertoire (61). The low expressing allele BF2*02:01 binds a wide variety of hydrophobic side chains from P_2_ and P_c_ in broad shallow pockets (**Figure 3**). In fact, binding two anchor residues with hydrophobic side chains is much like the human HLA-A2 molecules, but each subtype of HLA-A2 has a slightly different narrow P2-binding pocket that precisely fits a small subset of hydrophobic side chains (65–68). However, it seems likely that position 5 of the peptide is also a (very promiscuous) anchor residue, which has not been obvious in the two existing structures which have Gly and Ala at position 5. While BF2*02:01 has Arg9 and Asp24 just like BF2*21:01, these residues do not contact the anchor residues of the peptide because the groove is narrow, due to residues on the α-helices with large side chains in positions where BF2*21:01 has small residues. Another example of this mechanism for promiscuity is BF2*14:01 (**Figure 3**), which has a broad but deep pocket which accommodates larger hydrophobic residues at P2, again with an extremely promiscuous pocket for peptide position 5, along with an unprecedented cluster of acidic residues that bind one or more basic amino acids at the end of the peptide.

A third mechanism for promiscuous binding is illustrated by BF2*12:01 (47) (**Figure 2**), which is actually a fastidious molecule binding octamer peptides with stringent binding requirements at P5 and Pc (as well as having a promiscuous binding pocket for P2). However, this was the first reported chicken class I structure that showed a peptide extending out of the groove at the C-terminal end (although one was found for BF2*14:01 but not published). The key structural feature allowing this C-terminal overhang was noticed early on in chickens (and actually all non-mammalian vertebrates) (69, 70): an arginine at position 84 (HLA-A2 numbering) instead of the nearly invariant tyrosine found in nearly all classical and some non-classical class I molecules in mammals. Tyr84 is the residue that interacts with the C-terminal carboxyl group of the peptide and blocks off the C-terminal end of the groove in mammalian class I structures, but the equivalent position in mammalian class II molecules is in fact an arginine that facilitates the peptide leaving the groove. There are some reports of peptides hanging out of the groove for human class I molecules, but in these cases there are significant shifts in the conformation of the groove and a ten-fold loss in affinity (71–75), unlike in BF2*12:01. Peptides extending out of the groove are rare for the alleles from the so-called standard haplotypes derived from egg-layer chickens, but are found frequently for many alleles newly described for commercial meat-type (broiler) chickens. The crucial point is that such overhanging flanking residues in mammalian class II molecules can be recognised by T lymphocytes (76–78), with the inference that such overhangs for chicken class I molecules lead to a wider T cell response. However, there are no structures reported to illustrate the range of C-terminal overhangs in alleles from meat-type chickens, nor is there any evidence that these overhangs are recognised by chicken T cells, so these questions remain for future investigation.

**Figure 4:**
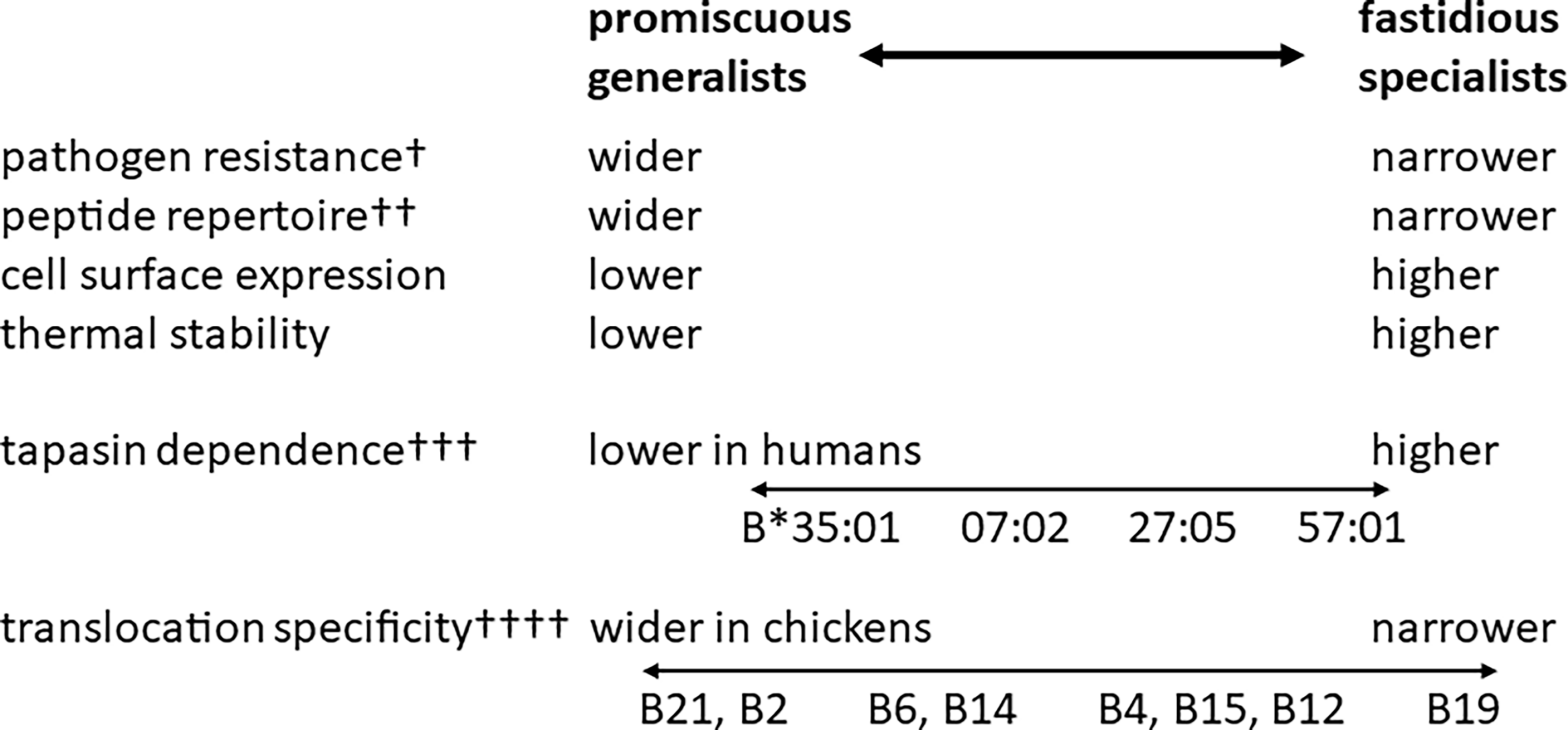
The hierarchies of class I alleles in humans and chickens have many similarities (pathogen resistance, peptide repertoire, cell surface expression and thermal stability), but they differ in some aspects (peptide translocation specificity is monomorphic in humans but polymorphic in chickens) and there is still much work to do to confirm the model (such as the role of tapasin in chickens). †, the ability of promiscuous human alleles to provide general protection against a variety of pathogens has not been rigorously tested; ††, the range of peptide repertoire is much broader in chickens than in humans; †††, tapasin dependence in chickens in not well understood; ††††, translocation specificity of human TAPs is monomorphic and much wider than the most promiscuous TAP alleles in chickens. HLA-B*57:01, B*27:05, B*07:02 and B*35:01 are human class I alleles, whereas B2, B4, B6, B12, B14, B15, B19 and B21 are chicken MHC haplotypes, each of which has a dominantly-expressed class I molecule (BF2). Figure based on references (22, 58).

The inverse correlation of peptide repertoire and cell surface expression level is part of a suite of properties, including resistance to Marek’s disease, TAP translocation specificity and thermostability (**Figure 4**), possibly indicating tapasin-dependence (56). Pulse-chase experiments show that amounts of promiscuous and fastidious class I heavy chains are roughly the same inside the cell, but that the movements of the class I molecules to cell surface differ, which is consistent with differences of TAP and tapasin alleles in the peptide-loading complex (53). The co-evolution between TAP and class I genes is supported by the peptide-translocation specificity of B21 cells, which is much wider than TAPs from haplotypes with fastidious BF2 molecules and indeed wider than the peptide-binding specificity of BF2*21:01 as assessed by refolding *in vitro* (53). The promiscuous class I molecules are less thermostable (53), correlating with the ease of refolding *in vitro*, which in humans is correlated with tapasin-independence (20, 61, 79).

A final point is that BF1 molecules are expressed by most chicken MHC haplotypes, although at low levels (43, 45). Sequence studies from over 200 chicken haplotypes show that many BF1 alleles have very similar sequences in the peptide-binding groove, with a His9 and Asp24 reminiscent of BF2*21:01. Given the wide range of peptide-translocation specificities among chicken MHC haplotypes, it is likely that most BF1 alleles are promiscuous so that they can find peptides no matter what (19). However, this point again remains to be shown. For completeness, the latest functional data suggests that the BF2 molecule is the major ligand for cytotoxic T lymphocytes, while the BF1 may be primarily a ligand for NK cells, and indeed has a sequence motif on the α1 helix like that found for one of the two subtypes of HLA-C in humans (80–82).

## Generalists and specialists: A concept that grew from peptide motifs and structures of chicken class I molecules

The existence of a hierarchy of BF2 alleles for a suite of properties prompted an examination of disease resistance in the literature for chicken MHC haplotypes as well as human class I alleles. In almost all of the published studies, chickens with a promiscuous class I molecule were found to be more disease resistant than chickens with a fastidious haplotype, including Marek’s disease, Rous sarcoma, infectious bronchitis and influenza (20). Even for a study comparing fastidious haplotypes for resistance to Rous sarcoma, the one with a motif that predicted the most peptides was the most resistant (43). A particularly impressive example sequenced the BF2 genes from dead and live indigenous chickens after a field outbreak of influenza in Thailand (83), with reanalysis showing that almost all of those with a promiscuous BF2 allele (B2 and B21) survived as homozygotes or heterozygotes, while most of those with only fastidious haplotypes died (20, 21). Since the haplotypes with promiscuous BF2 molecules were generally protective against a variety of economically-important pathogens, they were considered to act as generalists.

Only a few articles were found in the literature that compared disease associations in humans to some measure of peptide repertoire (20, 61). One examined four HLA-B alleles, comparing the number of self-peptides predicted to bind with the speed of progression from human immunodeficiency virus (HIV) infection to frank acquired immunodeficiency syndrome (AIDS), finding an inverse correlation between speed of progression and size of peptide repertoire (84). Said in another way, the elite controllers HLA-B57:01 and HLA-B27:05 are fastidious while the faster progressors HLA-B*07:02 and HLA-B*35:01 are promiscuous. Another study compared 27 HLA-A and HLA-B alleles for number of Dengue virus peptides predicted to bind as well as predicted affinity, finding that these measures were inversely correlated (85). HLA-B57:01, HLA-B*07:02 and HLA-B*35:01 were among those alleles tested and showed the same rank order as the previous study. Finally, a paper examining tapasin-dependence of 50 HLA-B alleles again had the same rank order for the four alleles (86), with the more tapasin-dependent and more stable alleles turning out to be the fastidious alleles (20, 56, 61).

As a result of these data compiled from the literature, flow cytometry was performed on lymphocytes and monocytes of suitable HLA-B homozygous individuals using three independent monoclonal antibodies, finding that expression level varied and was inversely correlated with peptide repertoire just as in chickens, but was directly correlated with tapasin-dependence and resistance to AIDS (61). For these two elite controller alleles, it was known that their fastidious peptide motifs focused on special HIV peptides that could be changed to avoid the immune response, but at the price of much lowered virus replication (87, 88). Given that these elite controller alleles bound special peptides for resistance, they could be considered as specialists to protect from particular pathogens.

Taken together, these data led to the hypothesis of generalist and specialist alleles (20, 61), in which a few promiscuous generalists would protect a population from a range of common pathogens, but a new or particularly nasty pathogen might be best resisted using a fastidious specialist. Further searching revealed a study in which a chicken haplotype with an allele now known to be fastidious protected from one but not another strain of RSV, while another haplotype with an allele now known to be somewhat promiscuous did the reverse (21, 89). Another study examined 96 HLA-A, B and C alleles for tapasin-dependence, showing large differences and finding that HIV progression to AIDS was slowest for tapasin-independent HLA-B alleles, as long as elite controllers were removed from the analysis (79).

Thus, for the comparisons available, the hierarchies of class I alleles in both humans and chickens have generalists and specialists at either end (**Figure 4**), suggesting that the correlations and the importance of peptide repertoire in disease resistance are fundamental properties of class I molecules. However, it appears that the hierarchy is wider in chickens. The most promiscuous chicken class I molecule is far more promiscuous than the most promiscuous human class I molecule, while most of the fastidious chicken class I molecules have three positions in which only one or a few amino acids are tolerated as opposed to the two anchors usually described for human motifs (43, 61). A computational paper extends some of the ideas about generalists by predicting peptides bound to human class II molecules, but the authors suggest that the number of different pathogens in an environment is the driving force for MHC alleles with promiscuous binding (90).

## A new frontier: The peptide motifs and structures of chicken class II molecules

In comparison to class I molecules, there are few reports describing classical class II molecules of chickens, and nothing for any other vertebrate species outside of mammals. Some differences in cell surface expression level and in DM-dependence of chicken class II molecules have been found, but the most progress has been made by biochemistry and structural studies (91, 92).

**Figure 5:**
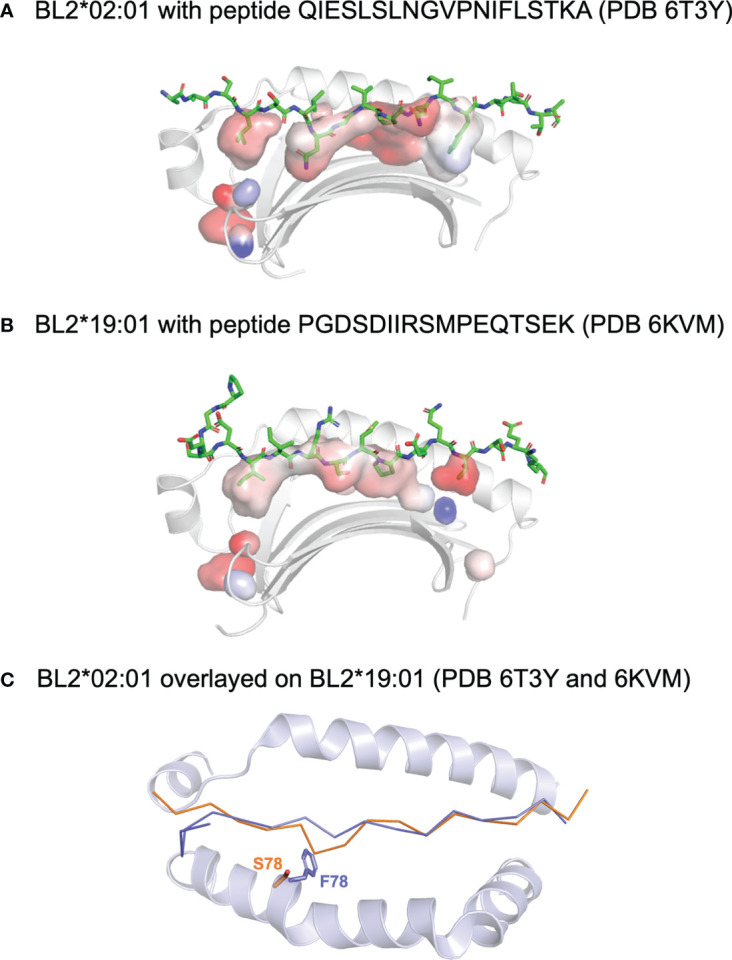
Peptides bound to chicken class II molecules. Side views of the structures (with the β1 helix removed) of **(A)** BL2*02:01 with peptide QIESLSLNGVPNIFLSTKA (PDB 6T3Y) and **(B)** BL2*19:01 with peptide PGDSDIIRSMPEQTSEK (PDB 6KVM). Peptide displayed with side chains (sticks in PyMol), class I molecules as ribbons (cartoon in PyMol), and with surface of the peptide binding groove to show pockets (with transparency). **(C)** Top down view of the superimposed structures, with the main chain of the peptide and the α-helices as cartoon except for the side chains of the key residues at position 78, showing that F78 of BL2*19:01 allows only the typical nonamer core, while smaller S78 of BL2*02:01 enforces a crinkle in the peptide (colours of position 78 and peptide match). Figure based on reference (98).

A structure of the dominantly-expressed class II molecule from the B19 haplotype, BL2*19:01, was determined (93) bound to a self-peptide identified by another group (94, 95) (**Figure 5**). As expected from sequence comparisons, most of the structure was similar to the many structures determined for mammals. A few small sequence differences with mammals that affect structural features were highlighted, including a four amino acid insertion in the α-chain that lengthens the contact between the first β-strands of the α1 and β1 domains. In addition, the amino acids in certain positions preclude a salt-bond between the α-helices and do not support a hydrogen bond network with DM found in mammals. In contrast, peptide-binding was largely conserved with mammals, including many of the residues that form hydrogen-bonds with the nonamer peptide core in a polyproline helix conformation. The residues that mediate interaction with CD4 in mammals were highly conserved, but it was pointed out that the chicken CD4 sequence has a deletion compared to mammals, so the actual mode of binding may differ.

A second structure of a dominantly-expressed class II molecule was reported for the B2 haplotype, BL2*02:01, in complex with an MDV peptide determined by immunopeptidomics of infected bursal B cells (96) (**Figure 5**). The key finding was that a decamer peptide core was found, unprecedented in all the many class II structures to date. The reason for the longer core bound to the groove was a crinkle in the peptide at peptide position P4, due entirely to a polymorphic position in a β-strand of the β1 domain, which is Ser in BL2*21:01 but large residues in nearly all class II molecules, including Phe in BL2*19:01. The fact that the two structures are from MHC haplotypes that confer resistance versus susceptibility leads to the question whether the length of the core could be another feature involved in response to this iconic pathogen. In fact, immunopeptidomics revealed that only four of the more than 100 genes of MDV were responsible for the vast majority of the peptides presented by the infected bursal B cells, unlike the reports for other herpesviruses infecting mammalian cells. Similar results found for three other chicken MHC haplotypes (B15, B19 and B21) may suggest an underlying mechanism for presenting peptides from just a few genes.

## Conclusions

From the first structure of a human class I molecule by Bjorkman, Wiley and their colleagues (97), X-ray crystallography and now cryo-electron microscopy have played a crucial role in understanding how mammalian MHC molecules present peptides to lymphocytes (98–106). However, there is much evidence to show significant differences among vertebrates in the structure, function and evolution of MHC molecules and the molecular pathways that support them (19). Thus far, the most detailed picture is for chicken class I molecules, but studies of chicken class II molecules and of MHC molecules throughout the jawed vertebrates have begun. However, there is no information yet about the interaction of the MHC molecules of chicken (or any other non-mammalian vertebrate) with TAP and tapasin in the peptide-loading complex, TAPBPR and TAPBPL, DM, CD4, T cell receptors or NK cell receptors. Together with careful studies at the levels of biochemistry, cell biology, cellular immunology, animal disease and population genetics, such structural studies will allow the evolution of the MHC and the adaptive immune system to be much better understood.

## Author contributions

SH and JK wrote the manuscript and made the figures.

## Funding

JK is a recipient of an Investigator Award from the Wellcome Trust (110106/Z/15/Z) and a project grant from the BBSRC (BB/V000756/1). SH is funded by the project grant from the BBSRC (BB/V000756/1). Our original uninvited articles can be supported for Open Access by the Wellcome Trust and BBSRC, as administered and agreed by the University of Edinburgh Open Access office.

## Acknowledgments

We thank the Wellcome Trust (Investigator Award 110106/A/15/Z) and the Biotechnology and Biological Sciences Research Council (BBSRC project grant BB/V000756/1) for support, and Lan Huynh and Magda Migalska for critical reading. For the purpose of open access, the author has applied a Creative Commons Attribution (CC BY) licence to any Author Accepted Manuscript version arising from this submission.

## Conflict of interest

The authors declare that the research was conducted in the absence of any commercial or financial relationships that could be construed as a potential conflict of interest.

## Publisher’s note

All claims expressed in this article are solely those of the authors and do not necessarily represent those of their affiliated organizations, or those of the publisher, the editors and the reviewers. Any product that may be evaluated in this article, or claim that may be made by its manufacturer, is not guaranteed or endorsed by the publisher.
